# Enhanced avidity from a multivalent fluorescent antimicrobial peptide enables pathogen detection in a human lung model

**DOI:** 10.1038/s41598-019-44804-0

**Published:** 2019-06-10

**Authors:** Ahsan R. Akram, Nicolaos Avlonitis, Emma Scholefield, Marc Vendrell, Neil McDonald, Tashfeen Aslam, Thomas H. Craven, Calum Gray, David S. Collie, Andrew J. Fisher, Paul A. Corris, Timothy Walsh, Christopher Haslett, Mark Bradley, Kevin Dhaliwal

**Affiliations:** 1 EPSRC IRC PROTEUS Hub, Centre for Inflammation Research, Queen’s Medical Research Institute, University of Edinburgh, Edinburgh BioQuarter, 47 Little France Crescent, Edinburgh, EH16 4TJ United Kingdom; 2 EaStCHEM, The University of Edinburgh School of Chemistry, Joseph Black Building, West Mains Road, EH9 3FJ Edinburgh, United Kingdom; 3 Clinical Research Imaging Centre, Queen’s Medical Research Institute, Edinburgh BioQuarter, University of Edinburgh, 47 Little France Crescent, Edinburgh, EH16 4TJ United Kingdom; 4 The Roslin Institute and R(D)SVS, The University of Edinburgh, Easter Bush Veterinary Centre, Roslin, Midlothian United Kingdom; 5 Institute of Transplantation, Newcastle University, Freeman Hospital, High Heaton, Newcastle upon Tyne, NE7 7DN United Kingdom

**Keywords:** Imaging and sensing, Infectious-disease diagnostics

## Abstract

Rapid *in situ* detection of pathogens coupled with high resolution imaging in the distal human lung has the potential to provide new insights and diagnostic utility in patients in whom pneumonia is suspected. We have previously described an antimicrobial peptide (AMP) Ubiquicidin (fragment UBI_29–41_) labelled with an environmentally sensitive fluorophore that optically detected bacteria *in vitro* but not *ex vivo*. Here, we describe further chemical development of this compound and demonstrate that altering the secondary structure of the AMP to generate a tri-branched dendrimeric scaffold provides enhanced signal *in vitro* and *ex vivo* and consequently allows the rapid detection of pathogens *in situ* in an explanted human lung. This compound (NBD-UBI_dend_) demonstrates bacterial labelling specificity for a broad panel of pathogenic bacteria and *Aspergillus fumigatus*. NBD-UBI_dend_ demonstrated high signal-to-noise fluorescence amplification upon target engagement, did not label host mammalian cells and was non-toxic and chemically robust within the inflamed biological environment. Intrapulmonary delivery of NBD-UBI_dend_, coupled with optical endomicroscopy demonstrated real-time, *in situ* detection of bacteria in explanted whole human Cystic Fibrosis lungs.

## Introduction

Pulmonary bacterial and fungal infection is a common and often fatal event^1,2^ and pneumonia is a foremost differential diagnosis in ventilated and immunocompromised patients who develop rapid respiratory deterioration^3^. Severe pneumonia, the development of ventilator associated pneumonia (VAP) and adult respiratory distress syndrome (ARDS) all cause significant morbidity and mortality and are responsible for a large proportion of healthcare costs^4–6^. Current management algorithms^7,8^ include the retrieval of bronchoalveolar lavage (BAL) or endotracheal sampling prior to empirical broad-spectrum antimicrobials, but all methods have variable sensitivity, are prone to contamination and confer significant time delays for microbial results.

Optical endomicroscopy (OEM) is a promising and attractive diagnostic platform^9–12^ and our work has demonstrated the utility to delineate gram-negative bacteria in mechanically ventilated patients *in situ*^13^ through an approach involving topically administered microdosed compounds with fiber based OEM. Furthermore, we have previously reported^14^ on the antimicrobial peptide (AMP) Ubiquicidin (UBI_29–41_) demonstrating that the linear fragment coupled with a fluorophore is insufficient for bacterial imaging in *ex vivo* lungs due to rapid degradation, despite being utilised as a nuclear medicine imaging agent^15^.

Here, we describe further development of this compound, engineering a multivalent (tri-branched) form, while utilising a modified portion of the bacterial binding fragment (UBI_29–41_) of the 59 amino acid sequence in which the metabolically labile methionine residue in replaced with a norleucine analogue. The arms of the trivalent scaffold were capped with the environmentally sensitive fluorophore, 7-nitrobenz-2-oxa-1,3-diazole (NBD)^16^ that self-quenches any residual fluorescence within the aqueous environment and then generates excellent signal-to-noise ratios when dispersed in the distal human lung, with fluorescent amplification only upon entry into the hydrophobic environment of the bacterial membrane. When compared to its’ monovalent equivalent, NBD-UBI_dend_ selectively labelled a diverse panel of pathogenic bacteria and *Aspergillus fumigatus*, and remained chemically stable in the complex environment of the inflamed and injured lung. It detected bacteria in an *ex vivo* large animal model of bacterial burden and in infected explanted cystic fibrosis (CF) human lungs.

## Results

### NBD-UBI_dend_ specifically labels bacteria and fungi with high signal-to-noise ratio over inflammatory cells

**Figure 1:**
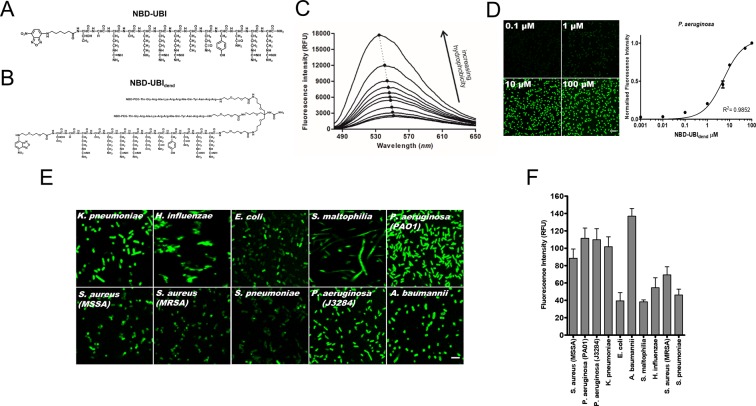
NBD-UBI_dend_ displays fluorescence amplification in hydrophobic environments and labels a diverse panel of clinically relevant pathogens in a concentration dependent manner. (**A**) The structure of NBD-UBI (monomer). (**B**) The structure of NBD-UBI_dend_ (multivalent construct). (**C**) NBD-UBI_dend_ demonstrates fluorescence amplification in increasing concentrations of *t*-butanol mimicking a hydrophobic environment (representative plot, n = 3). (**D**) Fluorescence quantification of *P*. *aeruginosa* imaged on a benchtop confocal microscope in the continual presence of increasing concentrations of NBD-UBI_dend_. Images show representative images at denoted concentrations of NBD-UBI_dend_ (scale bar represents 5 μm). Each point on the graph represents the mean (+/− SEM) of three independent experiments where at least three fields of view were quantified with a single site non-linear fit of the data. (**E**) Representative confocal images of a panel of clinically relevant pathogens imaged in the continued presence of NBD-UBI_dend_ (5 μM), scale bar represents 5 μm. *P*. *aeruginosa* data includes both the laboratory strain (PA01) and a clinical isolate from a patient with VAP (J3284). (**F**) Quantification of fluorescent intensity of bacterial imaging on a benchtop confocal microscope. Data shows the mean (+/− SEM) of three independent experiments where at least three independent fields of view were assessed.

The multivalent peptide (NBD-UBI_dend_) comprising three modified monomeric UBI peptides, coupled to the environmentally responsive fluorophore NBD (NBD-UBI) (Fig. 1A) was synthesised by solid-phase peptide synthesis (Fig. 1B).

NBD-UBI_dend_ demonstrated fluorescent amplification *in vitro* with increasing environmental hydrophobicity (Fig. 1C). Bacterial labelling was demonstrated in a concentration dependent manner (Fig. 1D) and of a panel of clinically relevant lung pathogenic bacteria, including both Gram-positive and Gram-negative species (Fig. 1E) with varying labelling intensity (Fig. 1F).

**Figure 2:**
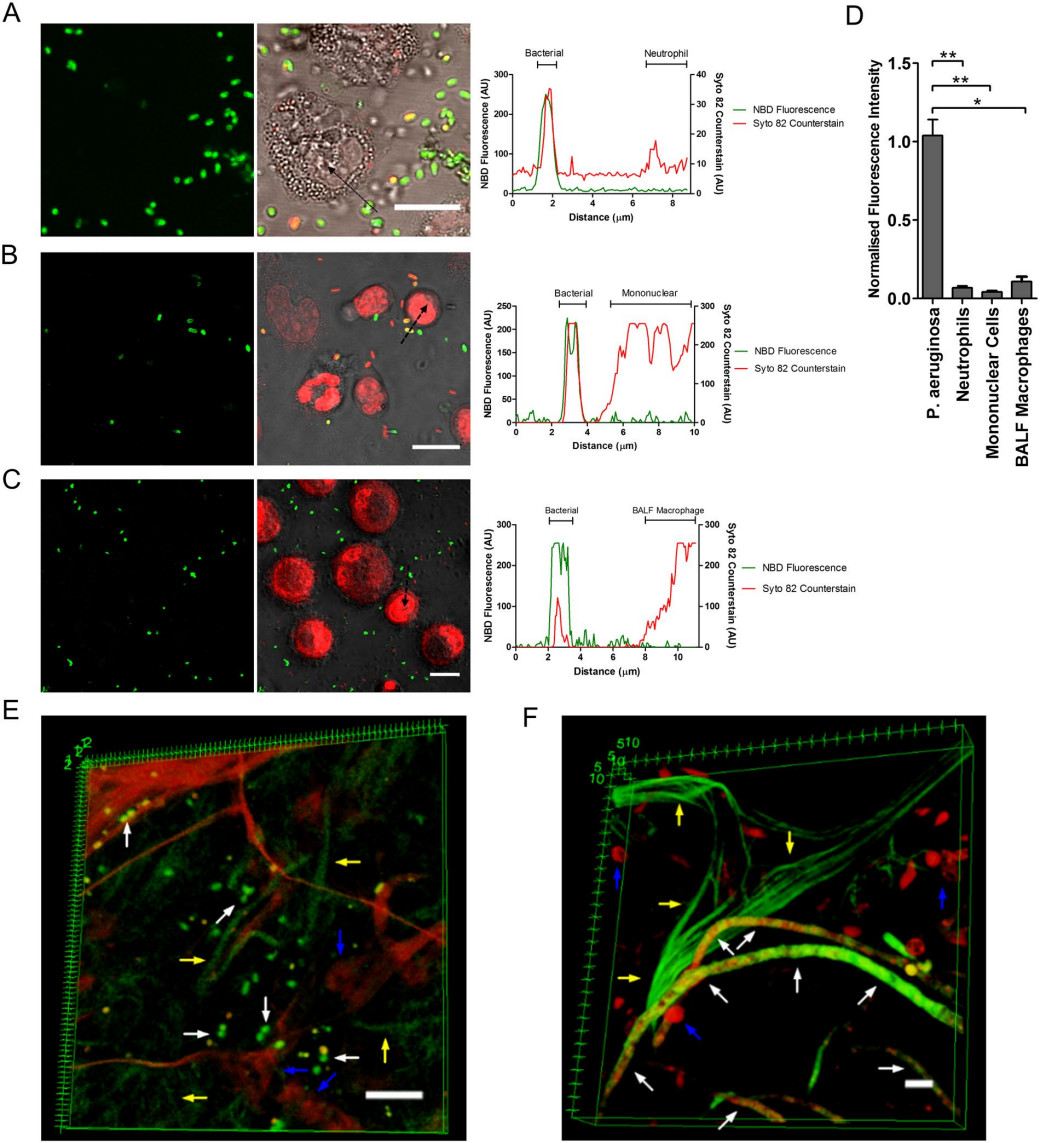
NBD-UBI_dend_ demonstrates selectivity for bacteria and *A*. *fumigatus* over human cells. (**A**–**C**) Co-culture of (**A**) freshly isolated human neutrophils, (**B**) freshly isolated human mononuclear cells and (**C**) human alveolar macrophages retrieved from bronchoalveolar lavage with *P*. *aeruginosa* imaged with NBD-UBI_dend_ 5 µM. Plot profiles quantify fluorescence across the dashed line in the NBD channel and counterstain (Syto-82) channel. Each panel shows representative images of at least three independent experiments for each cell type, scale bars represent 10 µm. (**D**) Quantification of benchtop confocal data demonstrating significantly lower fluorescence on neutrophils, mononuclear cells and BALF macrophages compared to bacteria. Bars show mean fluorescence (+/− SEM) from three independent experiments, where at least three fields of view were assessed. Analysis by Students t-test, *p < 0.05, **p < 0.01. (**E**,**F**) 3D reconstruction of benchtop confocal microscope imaging of (**E**) human alveolar tissue, incubated with *P*. *aeruginosa* and labelled with NBD-UBI_dend_ 5 µM and (**F**) human alveolar tissue, incubated with *A*. *fumigatus* and labelled with NBD-UBI_dend_ 10 µM. White arrows indicate specific labelling of bacteria or germinating fungal hyphae, whereas lung epithelial cells are not labelled (blue arrows). Yellow arrows indicate elastin autofluorescence present in the human lung. Images are representative of three independent experiments. Scale bars represent 10 µm.

Furthermore, to ensure the compound retains specify and does not label the inflammatory cell infiltrate observed with pneumonia, NBD-UBI_dend_ was co-cultured with freshly isolated primary human neutrophils, monocytes, lymphocytes and human alveolar macrophages (Fig. 2A–D) with no labelling seen. Confocal imaging was undertaken in the continued presence of the NBD-UBI_dend_ demonstrating the fluorescent amplification in bacteria. To further demonstrate selectivity in human lung tissue, bacterial labelling was demonstrated by confocal microscopy of human alveolar tissue^17^. Three-dimensional optical reconstructions confirmed bacterial-specific labelling within this complex environment (Fig. 2E).

NBD-UBI_dend_ also labelled the pulmonary pathogenic fungus *Aspergillus fumigatus* in human lung tissue (Fig. 2F). The pattern of labelling of *Aspergillus fumigatus*, imaged with OEM (Supplementary Fig. S1) was clearly discrete from bacteria, supporting the utility of NBD-UBI_dend_ and OEM to discriminate bacteria from *Aspergillus fumigatus*.

### NBD-UBI_dend_ demonstrates high avidity, stability when compared to its linear fragment and is non-toxic

**Figure 3:**
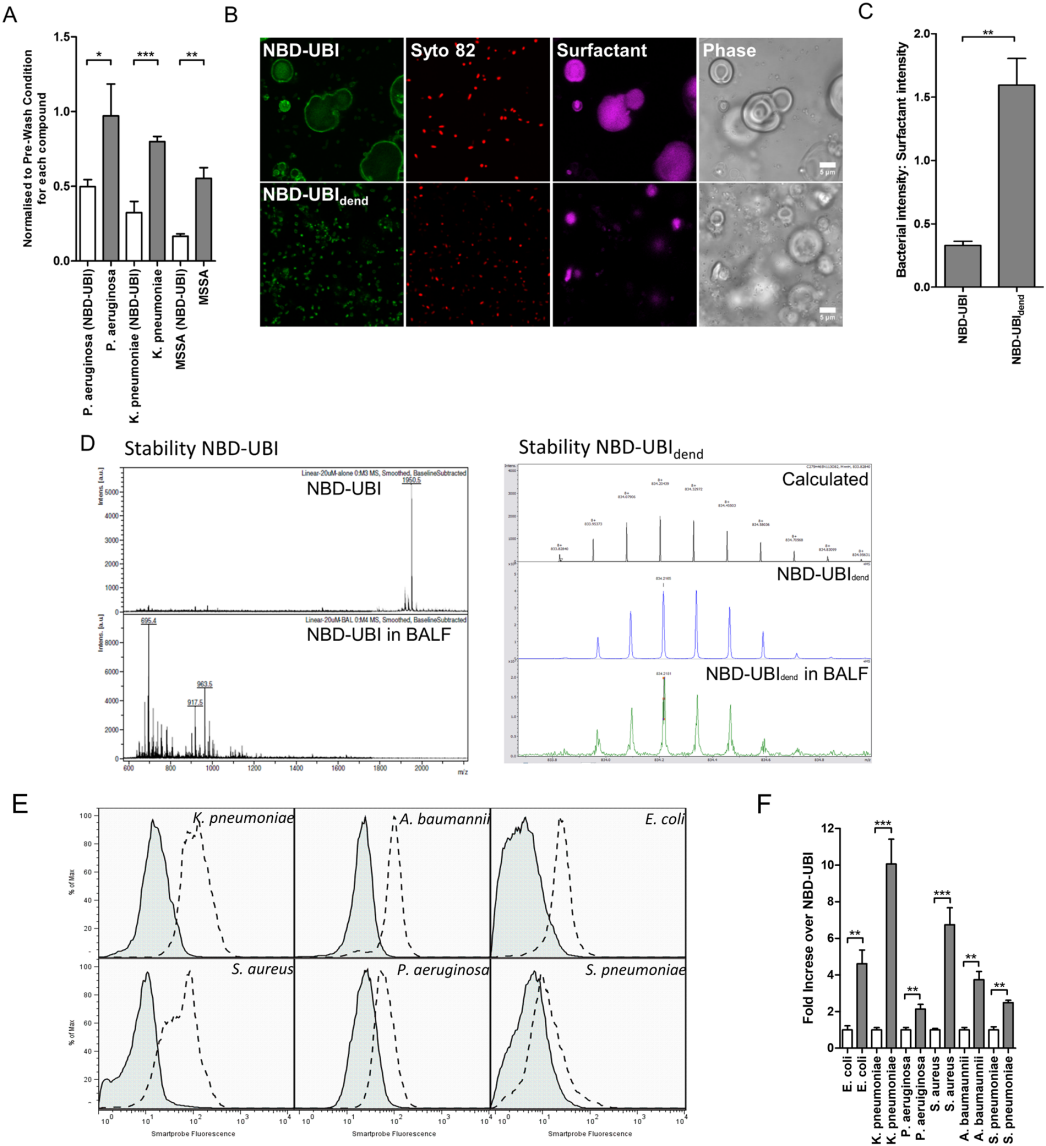
NBD-UBI_dend_ has greater affinity and demonstrates improved signal-to-noise when labelling bacteria in pulmonary surfactant than the linear NBD-UBI and shows stability in ARDS BALF. (**A**) Quantification of retained fluorescence following a wash and re-imaging on a benchtop confocal microscope. Each bacterial strain and compound were normalised to their pre-wash fluorescence intensities (bacteria were incubated with NBD-UBI at 15 μM or equimolar concentration of NBD-UBI_dend_ at 5 μM). Bars represent means (+/− SEM) of independent experiments where at least three fields of view were assessed, and analysed by a Student’s t-test, *p < 0.05, **p < 0.01, ***p < 0.001. (**B**) Representative images of *P*. *aeruginosa* imaged in the presence of synthetic pulmonary surfactant. Green panels show either NBD-UBI 10 μM or NBD-UBI_dend_ 3.3 μM with red panels showing Syto82 counterstaining, purple panels showing synthetic surfactant and phase contrast images demonstrating surfactant vesicles. (**C**) Fluorescence quantification of *P*. *aeruginosa* imaged with NBD-UBI at 10 μM or NBD-UBI_dend_ at 3.3 μM in the presence of fluorescently labelled high-density surfactant vesicles. Data represents the mean fluorescence of the NBD channel on bacteria compared to surfactant and show a significantly higher bacteria:surfactant fluorescence intensity for NBD-UBI_dend_ than NBD-UBI. Bars represent means (+/− SEM) of three independent experiments where at least three fields of view were assessed, analyses by Student’s t-test, **p < 0.01. (**D**) Stability of compounds in saline and ARDS BALF demonstrating breakdown of NBD-UBI in ARDS BALF when analysed by MALDI-TOF MS (left panel) the presence of NBD-UBI_dend_ in ARDS BALF when assessed by FTMS (right panel). (**E**) Representative flow histograms for bacteria when incubated with NBD-UBI (grey histograms) at 15 μM or NBD-UBI_dend_ 5 μM (dotted line) demonstrating a greater fluorescence intensity at eqimolar dye concentrations. (**F**) Quantification of flow cytometry data for the monomeric form (white bars, normalised) and dendrimeric forms (grey bars) demonstrating between a 2–10 fold increase in fluorescence at eqimolar dye equivalents. Bars represent means (+/− SEM) from three independent experiments, analysis is by students t-test, **p < 0.01, ***p < 0.001.

NBD-UBI_dend_ demonstrated high avidity for bacteria as evidenced by retention of bacterial labelling despite washing steps *in vitro*. Compared to 15 μM of the analogous linear moiety (NBD-UBI), 5 μM of NBD-UBI_dend_ retained bacterial labelling after washing (Fig. 3A). Hydrophobic phospholipoprotein components of pulmonary surfactant have the potential to generate off-target fluorescence. In order to investigate this, synthetic surfactant was co-cultured with bacteria and imaged. NBD-UBI_dend_ labelled bacteria in the presence of synthetic surfactant with good signal-to-noise ratios (Fig. 3B,C). Chemical stability was assessed by incubating NBD-UBI_dend_ and NBD-UBI with bronchoalveolar lavage fluid (BALF) from ARDS patients in ICU and performing mass spectroscopic analysis. NBD-UBI was rapidly degraded in ARDS BALF, while NBD-UBI_dend_ remained intact (Fig. 3D). Furthermore, the multivalent peptide provided enhanced fluorescent labelling when compared to equimolar levels of the analogous monomeric construct NBD-UBI (Fig. 3E,F).

NBD-UBI_dend_ demonstrated no overt biological toxicity, evidenced by absence of erythrocyte hemolysis, and no preclinical *in vivo* toxicity: murine intratracheal instillation of high concentrations (over 700 times greater than the final intended human pulmonary dosing), resulted in no pulmonary inflammatory cell recruitment, pulmonary toxicity or systemic toxicity at early and late time-points (Supplementary Fig. S2A–D).

### NBD-UBI_dend_ rapidly detects bacteria with topical microdosed endobronchial delivery and OEM in a large animal *ex vivo* lung model

**Figure 4:**
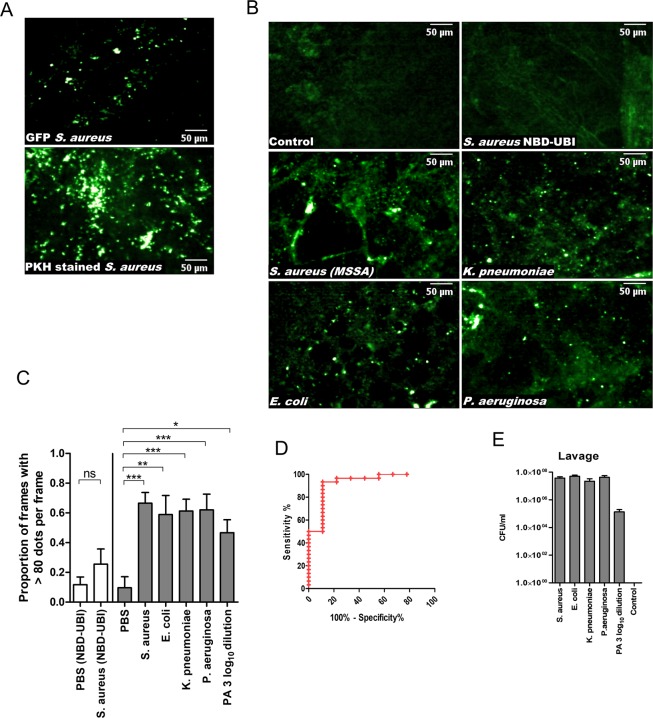
NBD-UBI_dend_ labels bacteria *in situ* in an *ex vivo* ovine lung model of pneumonia. (**A**) Panels show representative images of positive control of GFP *S*. *aureus* (upper) or PKH stained *S*. *aureus* (lower) instilled into an ovine pulmonary segment and imaged demonstrating a characteristic punctate fluorescent bacterial signal. (**B**) Representative images of OEM imaging where control segments (PBS vehicle control) and *S*. *aureus* segment with monomer NBD-UBI do not show characteristic punctate bacterial signal, whereas *S*. *aureus*, *K*. *pneumoniae*, *E*. *coli* and *P*. *aeruginosa* with NBD-UBI_dend_ (2 μM) demonstrate *in situ* bacterial labelling when the SmartProbe is applied topically in the distal alveolar space (see also Videos 1–3). (**C**) Image analysis algorithm of entire datasets following the removal of redundant frames. Bars represents the combined mean (+/− SEM) of the percentage of frames with over 80 detected dots per frame over the imaging sequence for individual experiments. White bars represent NBD-UBI (monomer) with no significant increase for *S*. *aureus* (n = 6) over PBS control segments (n = 6), ns = not significant. Grey bars demonstrates a significantly higher number of detected dots per frame for the bacterial segments (*S*. *aureus* n = 8, *E*. *coli* n = 6, *K*. *pneumoniae* n = 8, *P*. *aeruginosa* n = 8) when compared to control segment (n = 9) for NBD-UBI_dend_, *p < 0.05, **p < 0.01, ***p < 0.001 Students t-test. (**D**) Performance characteristics to distinguish control segments (PBS instilled, n = 9) or bacterial segments (comprising of *S*. *aureus*, *E*. *coli*, *K*. *pneumoniae* or *P*. *aeruginosa*, n = 30) with NBD-UBI_dend_ demonstrating an area under the curve (95% confidence interval) of 0.9259 (0.8169 to 1.035), p = 0.0001280. (**E**) BALF from ovine segments demonstrating clinically relevant bacterial yields, n = 4 for all segments except dilution of *P*. *aeruginosa* (PA) where n = 3. Bars represent mean (+/− SEM).

Using a previously reported model^13^ we evaluated NBD-UBI_dend_ against a diverse panel of clinically relevant pathogenic bacteria (Fig. 4). NBD-UBI_dend_ was able to label bacterial segments, over control segments and the linear NBD-UBI compound within a clinically relevant limit of detection (LoD) of 1 × 10^5^ CFU/mL bacteria retrieved on BALF (Fig. 4A–F and Videos 1–4). Monomeric NBD-UBI failed to detect bacteria (Fig. 4D) in this model.

### NBD-UBI_dend_ detects bacteria *in situ* in whole explanted human lungs

**Figure 5:**
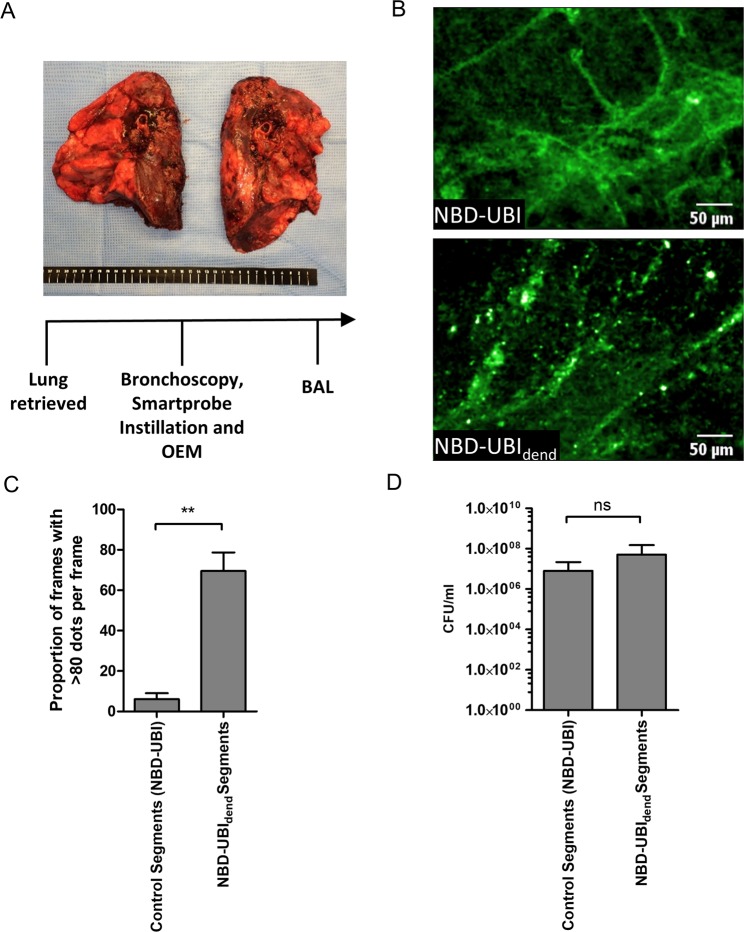
NBD-UBI_dend_ labels colonised bacteria *in situ* in whole explant Cystic Fibrosis (CF) Human Lungs. (**A**) Experimental procedure of CF lungs with bronchoscopy, Smartprobe instillation, OEM imaging and bronchoalveolar lavage retrieval. (**B**) Representative frames of segments instilled with NBD-UBI (6 µM) with minimal punctate signal (upper) and segment instilled with NBD-UBI_dend_ (2 µM) demonstrating the characteristic punctate signal associated with bacterial labelling. (**C**) Analysis of OEM videos with image analysis algorithm showing a significantly higher proportion of frames containing >80 dots per frame (bacterial infection). Bars represents the combined mean (+/− SEM) of the percentage of frames with over 80 detected dots per frame over the imaging sequence for individual segments, **p < 0.01, n = 3 CF patient explants. (**D**) Lavage counts from BALF retrieval for control, n = 3 from two CF lungs and NBD-UBI_dend_, n = 4 from three CF lungs. Bars represent mean (+/− SD) of counts.

To further support clinical utility, we performed bronchoscopy and delivery of NBD-UBI_dend_ in freshly-explanted whole lungs from patients with cystic fibrosis (CF) undergoing transplantation (Fig. 5A). The CF lungs were extremely damaged with large amounts of mucopurulent secretions. Discrete distal bronchopulmonary segments were instilled with NBD-UBI_dend_ or equimolar monomeric NBD-UBI (negative control). The characteristic bacterial signal of punctate twinkling speckles and also distinct and intense colonies were detected in the alveoli in the NBD-UBI_dend_ instilled segments, but not the monomeric instilled segments (Fig. 5B,C and Video 5). BAL confirmed the presence of pathogenic bacteria in all segments of the CF lung (Fig. 5D). This confirmed the ability of NBD-UBI_dend_ to rapidly detect bacteria *in situ* in the human lung using a microdosing regime and OEM.

## Discussion

Immediate-point-of-care bedside methodologies to enable clinicians to determine the presence or absence of bacteria or pathogenic fungi in the distal lung are required. Clinicians are faced with significant uncertainty in relation to triggers to commence antibiotic treatment^7^, the choice of agents^18^ and especially when to de-escalate therapy^19^. These issues are barriers to effective antibiotic stewardship because of the association between delayed and inadequate antibiotic therapy and adverse clinical outcomes^20^.

Optical imaging of bacteria is an emerging and attractive modality^21–23^ and utilising OEM with localised microdosed delivery of SmartProbes in distal bronchopulmonary segments potentially offers an immediate method for bacterial detection. This approach shows particular promise for the future as it can be multiplexed using fiber based systems capable of multispectral imaging^24,25^, used in combination with other SmartProbes for Gram-negative bacteria^13^ or inflammatory cells^26,27^ and compounds can be delivered directly into the imaging field of view^28^. Furthermore, with ongoing refinements to the image analysis algorithms through multiple methods^29–31^, automated readouts of the signals generated may help decision making and advance our understanding of the pathophysiology during suspected pneumonia.

UBI_29–41_, in its monomeric form, has been applied in human infection imaging in the form of radionuclide imaging with technetium^32^. We demonstrated that this monomeric scaffold was rapidly degraded in the inflamed bronchoalveolar compartment, and have demonstrated that a monomeric ligand remains ineffective for bacterial labelling in assays using fragments of human lung tissue^14^. In contrast, the trivalent dendrimeric scaffold afforded enhanced proteolytic stability, partitioning in surfactant and high avidity in the whole human lung.

Although this compound demonstrates promise for clinical utility, it now requires clinical translation to determine utility alongside other SmartProbes in patients with suspected pneumonia.

In summary, we have synthesized a multivalent fluorescent UBI analogue that demonstrates excellent optical properties for the detection by optical imaging platforms already in clinical use and with microdosed delivery selectively labels a range of pathogens *in vitro* and in an *ex vivo* ovine model. Importantly, we have also shown that this novel technology can specifically detect bacteria in the harsh environment of freshly explanted human lungs from patients with CF.

## Materials and Methods

### Ethics statement

All experiments using human samples were performed following approval of the appropriate regional ethics committee (REC), with informed consent of the patients and in accordance with the relevant guidelines and regulations at the University of Edinburgh. BALF for alveolar macrophages: (East of Scotland Research Ethics Service REC no: 07/S1102/20), blood for isolation of neutrophils and mononuclear cells: (Lothian REC no: 08/S1103/38), human lung tissue: (East of Scotland Research Ethics Service REC no: 13/ES/0126) and explant Cystic Fibrosis Lung assessment: (NRES Committee North East-Newcastle & North Tyneside REC no: 11/NE/0291). Animal experiments were performed under UK Home Office Animals Scientific Procedures Act 1986 (Project License Number 60/4434) in accordance with the guidelines and regulations at the University of Edinburgh and following approval of experimental protocols by the Animal Welfare and Ethical Review Body at the University of Edinburgh. Ovine lungs were from ewes destined for cull and were euthanized under Schedule 1 of Animals (Scientific Procedures) Act 1986.

### Chemical synthesis and characterisation

The synthetic procedures and characterization data for all the chemical probes is described in the Supplementary Information.

### Bacterial culture

Bacterial strains were grown and counterstained as previously described^13,14^. The following bacterial strains were used: Methicillin Resistant *S*. *aureus* (ATCC 25923), Methicillin Sensitive *S*. *aureus* (ATCC 252), *S*. *pneumoniae* (D39 NCTC 7466), *H*. *influenzae* (Clinical Isolate), *K*. *pneumoniae* (ATCC BAA1706), *E*. *coli* (ATCC 25922), *A*. *baumannii* (J3433 Clinical Isolate), *S*. *maltophilia* (J3270 Clinical Isolate), *P*. *aeruginosa* (PA01- ATCC 47085 and J3284-clinical isolate) and GFP fluorescent *S*. *aureus* (RN6390-Gfp-EryR).

### Fungal culture

*Aspergillus fumigatus* (clinical isolate) was stored in potato glucose agar (Fluka Analytical, Gillingham, UK) at 4 °C. A wet harvest of conidia was performed into 1 mL of potato dextrose broth (Fluka Analytical, Gillingham, UK) and conidia were counted on a haemocytometer. 1 × 10^6^ conidia were added to 9 mL potato dextrose broth and grown overnight in a shaking incubator at 37 °C. Germinating hyphae were washed for confocal assays.

### Neutrophil and mononuclear cell isolation

Neutrophils and mononuclear cells were isolated from the peripheral blood of healthy human volunteers using dextran sedimentation and discontinuous Percoll gradients, as previously described^33^.

### MALDI-TOF and FTMS

NBD-UBI or NBD-UBI_dend_ were added to saline or pooled BALF from three patients with ARDS incubated for 30 min. ARDS BALF was retrieved from patients in ICU, as previously described^34^. Samples were passed through ZipTip (C-18, 0.2 µL) conditioned with 5 µL MeCN (with 0.1% TFA as an additive) followed by 20 µL of H_2_O. The ZipTip was loaded with the sample, washed and eluted with 5 µL of 80% aq. MeCN (with 0.1% TFA as an additive). Samples were analysed on a MALDI-TOF (PerSeptive Biosystems Voyager DE™STR MALDI-TOF) mass spectrometer, Applied Biosystems, Foster City, CA) or FTMS (Bruker Daltonics 12 T SolariX Fourier Transform Ion Cyclotron Resonance Mass Spectrometer (FT-ICR MS)).

### Emission spectra

The fluorescence emission of NBD-UBI_dend_ solutions were measured in increasing concentrations of t-butanol on a Synergy H1 Multi-Mode Spectrophotometer (BioTek, VT, US) upon excitation at 450 nm.

### Surfactant constituent synthesis

Surfactant vesicles were synthesised by dissolving 5 µg 1,2-Dipalmitoyl-sn-glycero-3-phosphocholine (DPPC) and 2.5 µg L-α-Phosphatidyl-DL-glycerol sodium salt (from egg yolk lecithin; PG) in 500 µl chloroform. The solution was evaporated under nitrogen to form a thin lipid film in a round bottom flask and subsequently rehydrated with PBS at 48 °C for 1 hour with continuous agitation at 750 rpm to generate multilammelar vesicles (MLV). MLVs were stained with Cellvue Claret dye (Sigma-Aldrich, St Louis, MO, USA) and diluted 1:4 for use in confocal experiments.

### Haemolysis assay

Performed as previously decribed^13^. Briefly, erythrocytes (resuspended to 20 vol % in PBS) were cultured with varying concentrations of NBD-UBI_dend,_ PBS (negative control) or 0.2% Triton X-100 (positive control) and incubated at 37 °C for 1 h. Wells were diluted with 150 µl of PBS, centrifuged and absorbance of supernatant read at 350 nm.

### Single dose intra-tracheal rodent study

Adult male CD-1 mice aged 8–12 weeks were given direct intra-tracheal administration of a single dose of NBD-UBI_dend_ (100 µg) in 50 µl PBS (equivalent to 300 µM) or PBS vehicle control. Animals were monitored and then sacrificed at 48 hours or 14 days (n = 3 per group per time point). Airway resistance was assessed via whole body plethysmography. Following necroscopy, bronchoalveolar lavage fluid (BALF) was harvested via 3 × 0.8 ml PBS flushes of the lung and cytospin slides were prepared and stained with Diff-Quick (Thermo Fisher Scientific). BALF cell types were quantified using a light microscope. Lung, liver and kidney were removed, fixed and paraffin-embedded. Slides were prepared and stained with hematoxylin and eosin.

### Confocal imaging and analysis

Imaging and analysis was conducted as previously reported^13,14^. ‘Green’ fluorescence (for NBD) was excited with a dedicated 488 nm line, Syto nuclear acid dyes were excited with a dedicated 543 nm line and Cellvue Claret labelled surfactant were excited with a dedicated 633 nm line. For affinity assays, following initial imaging, the fluid was aspirated from wells and two gentle washes with PBS were performed. The chamber was re-imaged on benchtop confocal.

### Flow cytometry

NBD-UBI or NBD-UBI_dend_ were incubated with bacteria for 5 minutes at 37 °C prior to analysis using BD FACSCalibur (Becton Dickenson, San Jose, CA, USA) flow cytometer capturing 50,000 of counterstained gated events. Analysis performed with FlowJo version 7.6.5 (TreeStar Inc., Ashland, OR).

### Ovine lung model and image analysis

The ovine model and analysis algorithm have been reported previously^13^. Briefly, surplus stock animals, which were destined for cull, had their lungs removed, which were ventilated and kept at 37 °C, humidity of 65% and ventilated using a Pressure Controlled Ventilator (Vivo PV 403, Breas Medical, Sweden). Segments were bronchoscopically identified and bacteria (or PBS control) instilled. After >1 hour segments were re-identified with subsequent SmartProbe instillation and OEM imaging at 488 nm excitation (Cellvizio Lab, Mauna Kea Technologies) using a 1.5 mm diameter S-1500 fiber (Mauna Kea Technologies), followed by BALF retrieval. Frame-by-frame analysis was undertaken^13^ after removal of redundant frames; frames were considered positive if there were >80 dots per frame. Data is presented as the proportion of frames over a video sequence containing >80 dots per frame.

### *Ex vivo* cystic fibrosis human lung

Whole explant CF lungs were received fresh from theatre (<1 hour) and main the bronchi were identified. Bronchoscopy and suction of the airways was performed prior to instilling NBD-UBI/NBD-UBI_dend_ into anatomically distinct distal bronchopulmonary segments. The bronchoscope was proximally wedged and the S-1500 fiber passed into distal segments. Following imaging in the distal lung (alveolar regions) as described above, a BAL was undertaken, sample was centrifuged at 200 g for 5 minutes to remove erythrocytes and cellular material and was plated for bacterial quantification.

### Statistical analysis

All experiments were performed at least three times unless otherwise stated and results expressed as mean ± SEM. Data was analysed by Student’s t-test or ANOVA, significance was determined as p < 0.05 (GraphPad Prism version 5.01 for Windows, GraphPad Software, San Diego California USA).

## Supplementary information

Supplementary Information

Video 1

Video 2

Video 3

Video 4

Video 5

## Data Availability

The data that support the findings of this study are presented in the published article and any additional data is available from the corresponding author upon reasonable request.
