# Supplementary material for: Enhanced avidity from a multivalent fluorescent antimicrobial peptide enables pathogen detection in a human lung model
Source: Sci Rep. 2019 Jun 10;9:8422. doi: 10.1038/s41598-019-44804-0 (PMC6557859; doi:10.1038/s41598-019-44804-0)
Supplement: Supplementary file 1 — Supplementary Information [file 41598_2019_44804_MOESM1_ESM.pdf]

Supplementary Information

Enhanced avidity from a multivalent fluorescent antimicrobial peptide enables pathogen detection in a human lung model.

Ahsan R Akram, Nicolaos Avlonitis, Emma Scholefield, Marc Vendrell, Neil McDonald, Tashfeen Aslam, Thomas H Craven, Calum Gray, David S Collie, Andrew J Fisher, Paul A Corris, Timothy Walsh, Christopher Haslett, Mark Bradley & Kevin Dhaliwal.

Supplementary Figures:

Supplementary Figure S1: ***A. fumigatus*** **imaged on a benchtop confocal and OEM demonstrating labelling of germinated hyphae……………………………………………………………………………………2**

Supplementary Figure S2: NBD-UBI$_{dend}$ demonstrates no membrane toxicity and has no acute (48 hours) or delayed (14 days) pulmonary or systemic toxicity when administered intratracheally……………………………………………………………………………………3

Supplementary Methods:

Chemical synthesis including Schemes M1 and M2, and Figures M1 and M2. ………………………………4

Supplementary references……………………………………………………………………………9

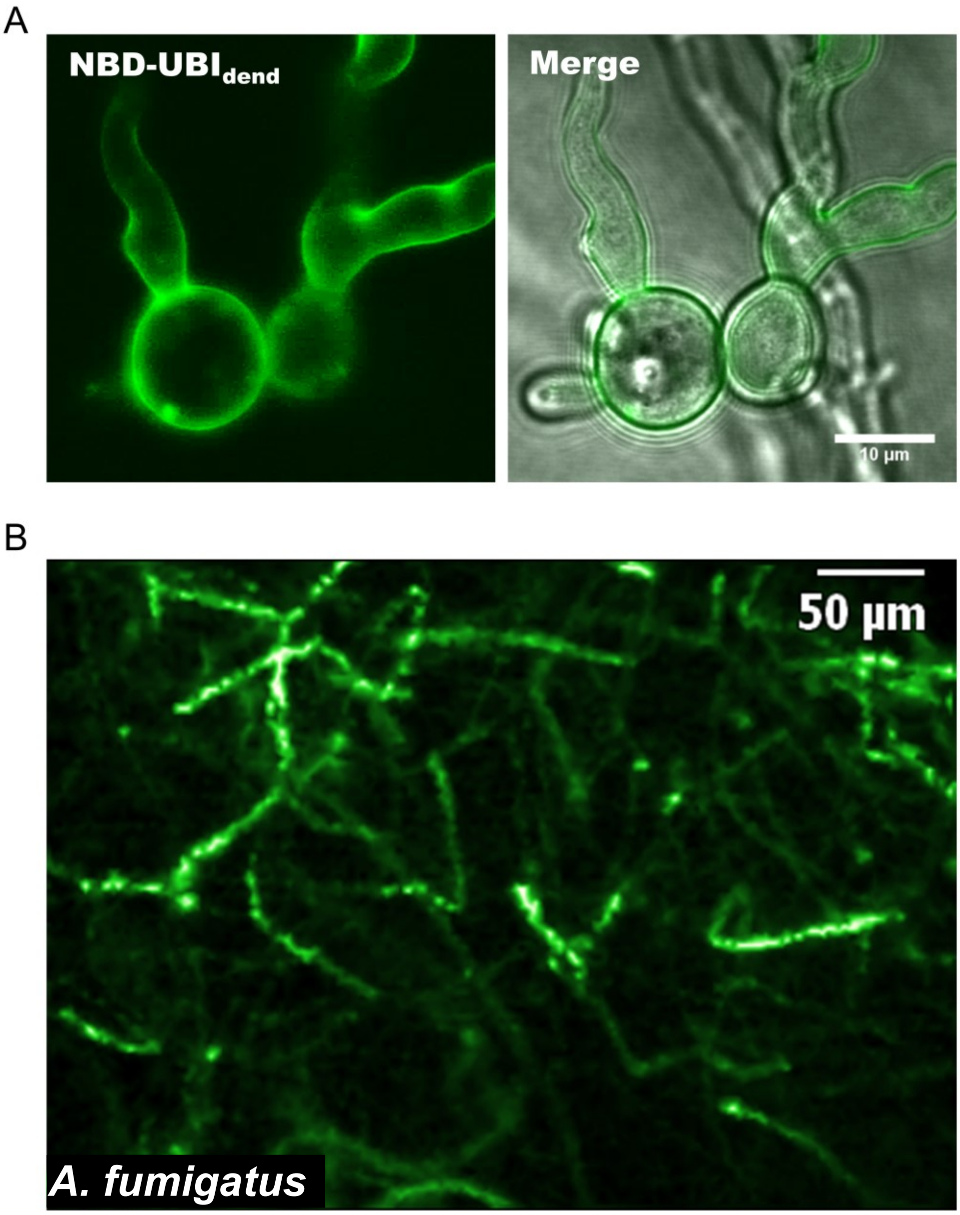

Supplementary Figure S1: A. fumigatus imaged on a benchtop confocal and OEM demonstrating labelling of germinated hyphae. A) Representative confocal image of membrane labelling of germinating *A. fumigatus* in the continued presence of NBD-UBI$_{dend}$ (5µM). Image on left represents the NBD fluorescence and image on right is merge with phase contrast. **B)** Representative still image of a frame from a video of *A. fumigatus* imaged with NBD-UBI$_{dend}$ via OEM demonstrating the characteristic hyphal labelling, which is distinct from the bacterial signal.

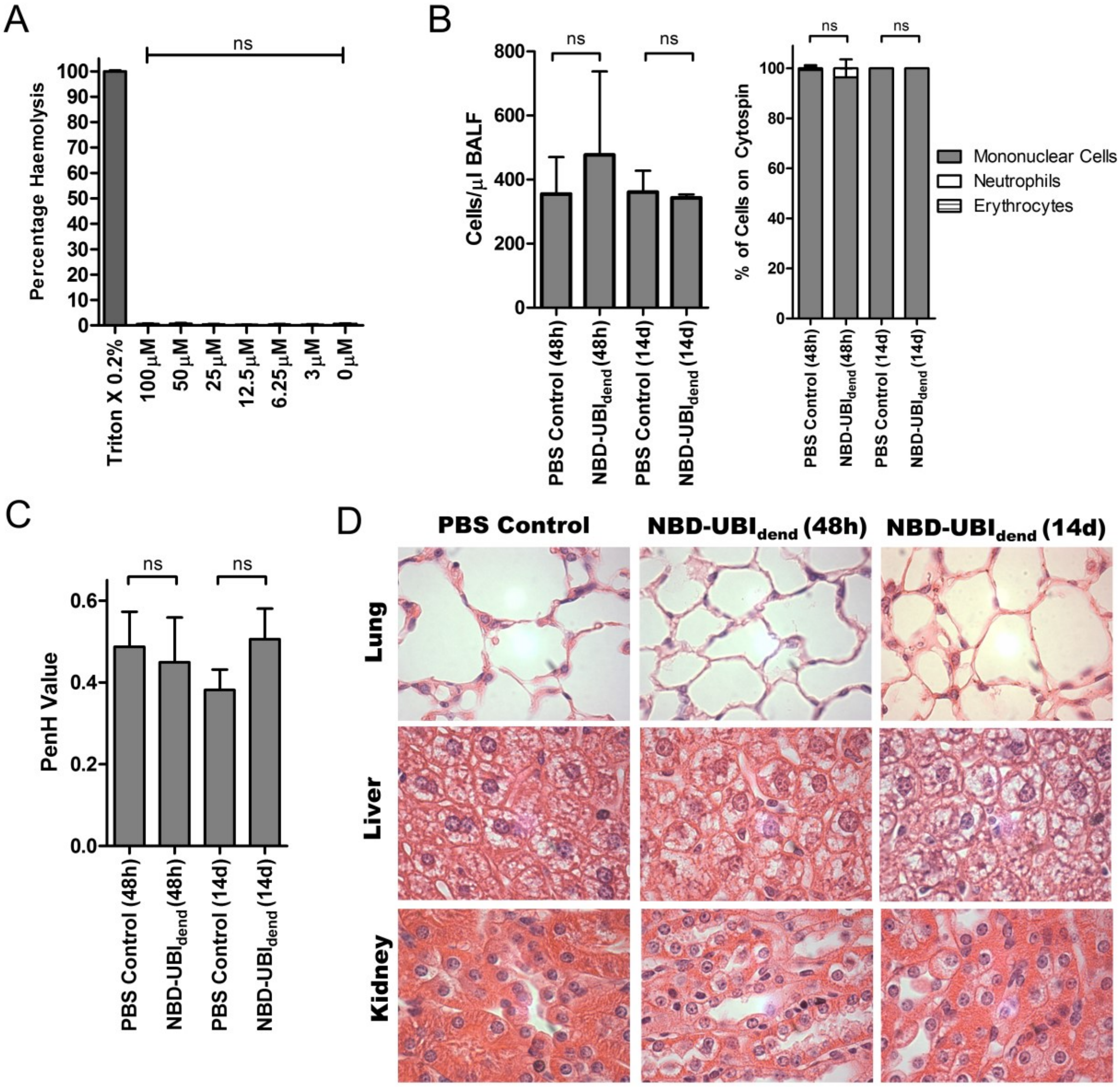

Supplementary Figure S2: NBD-UBI$_{dend}$ demonstrates no membrane toxicity and has no acute (48 hours) or delayed (14 days) pulmonary or systemic toxicity when administered intratracheally to mice. a) No red cell haemolysis obsereved for up to 100μM, n=3, bars represent mean (+/-SEM) of three independent experiments performed in duplicate. Positive control was 0.2% Triton-X and values corrected to represent 100% haemolysis for Triton-X. Statistical Analysis by ANOVA, ns=not significant. b) Murine BALF retrieved at 48 hours following intratracheal instillation of 100 micrograms of NBD-UBI$_{dend}$ (vs vehicle PBS control) demonstrates no cellular influx. Bars represent mean (+/-SD), n=3. c) Airways resistance as a surrogate of pulmonary function measured by PenH showed no pulmonary function changes, bars represent mean (+/-SD), n=3. d) Representative organ histology (x100) demonstrating no pathological findings in lung, liver or kidney tissue after intratracheal administration of NBD-UBI$_{dend}$.

Chemical synthesis

The isocyanate (**6**) was prepared as described in Scheme M1 in an overall yield of 15%, following a reported procedures (*1*).

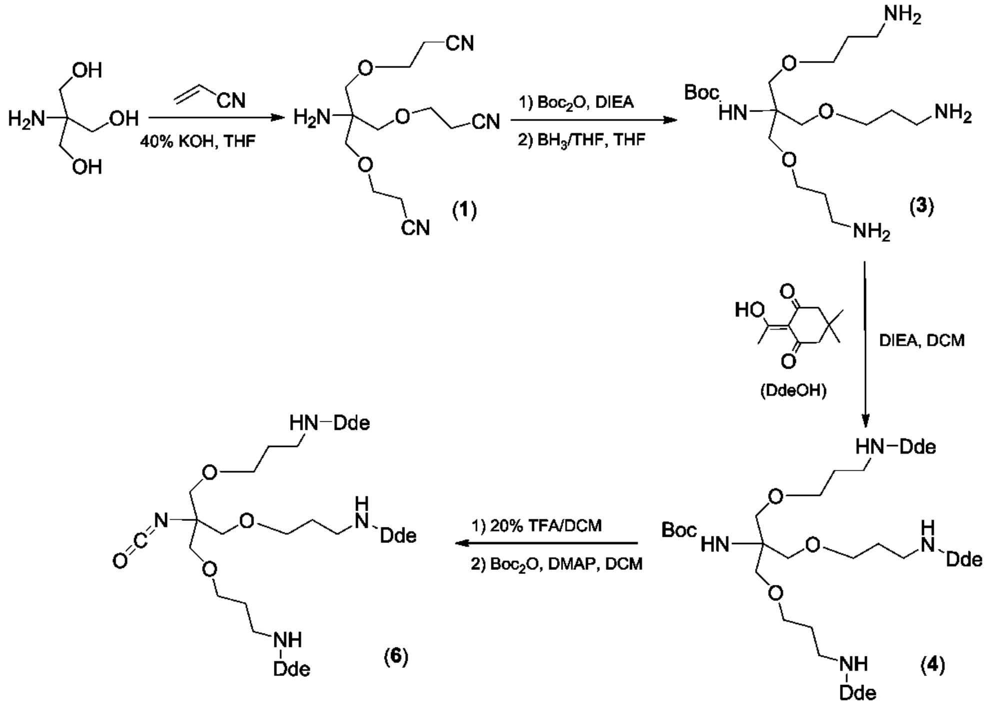

Scheme M1. Synthesis of the isocyanate (**6**).

NBD-UBI$_{dend}$ (Scheme M2) was synthesised on aminomethyl polystyrene resin (0.745 mmol/g, 1% DVB, 100-200 mesh) derivatized with an Fmoc-Rink Amide type linker (Scheme S3). The linker was loaded with isocyanate (**6**) to give the tri-branched scaffold (**7**). Following the removal of the Dde groups (2% hydrazine in DMF), the appropriate Fmoc-protected amino acids were coupled using conventional SPPS protocols followed by the coupling of 4-chloro-7-nitrobenzofurazan (NBD-Cl) .Final cleavage of the product was carried out using a cocktail of TFA:phenol:water:TIS (88:5:5:2).

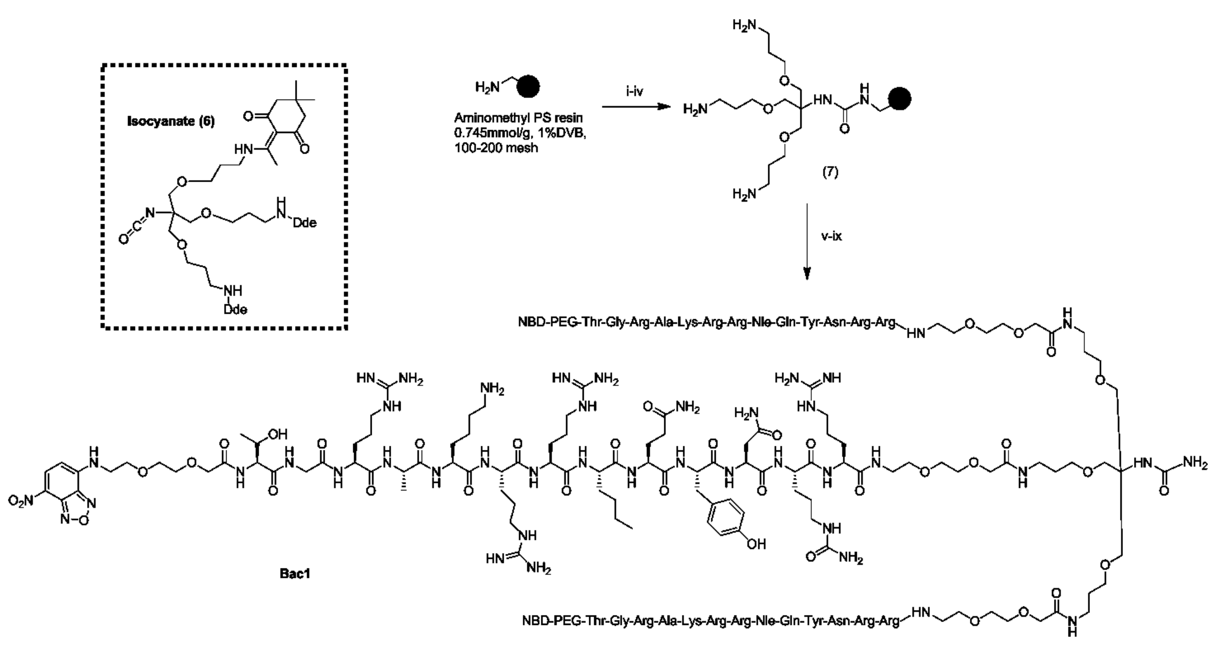

Scheme M2. Synthesis of **NBD-UBI$_{dend}$**. Reagents and conditions: i) Fmoc-Rink amide linker, Oxyma, DIC, DMF; ii) 20% piperidine/DMF; iii) Isocyanate (6), DIPEA, DMAP, DCM/DMF; iv) 2% Hydrazine/DMF; v) a) Fmoc-PEG-OH, Oxyma, DIC, DMF, b) 20% piperidine/DMF; vi) a) Fmoc-AA-OH, Oxyma, DIC, DMF, b) 20% piperidine/DMF; vii) a) Fmoc-PEG-OH, Oxyma, DIC, DMF, b) 20% piperidine/DMF; viii) NBD-Cl, DIPEA, DMF; ix) TFA:phenol:water:TIS (88:5:5:2). PEG: 8-amino-3,6-dioxaoctanoic acid, NBD-Cl: 4-chloro-7-nitrobenzofurazan, Dde: N-(1-(4,4-dimethyl-2,6-dioxocyclohexylidene)ethyl).

Fmoc-Rink amide polystyrene resin

4-[(2,4-dimethoxyphenyl)-(Fmoc-amino)methyl]phenoxyacetic acid (Rink amide linker) was attached to aminomethyl polystyrene resin (0.745 mmol/g, 1% DVB, 100-200 mesh). The Fmoc-Rink-amide linker (2.2 mmol, 3 eq) was dissolved in DMF (16mL) and ethyl oximinocyanoacetate (Oxyma) (2.2 mmol, 3 eq) was added, and the mixture was stirred for 10 min. *N,N'*-Diisopropylcarbodiimide (DIC) (2.2 mmol, 3 eq) was then added and the resulting mixture was stirred for further 5 min. The solution was added to aminomethyl polystyrene resin (0.745 mmol, 0.745 mmol/g, 1 eq) and shaken for 2 h. The resulting resin was washed with DMF (3×10 mL), DCM (3×10 mL) and MeOH (3×10 mL). The coupling reaction was monitored by a quantitative ninhydrin test (*2*).

Isocyanate coupling (7)

To the resin (400 mg, 0.30 mmol), pre-swollen in DCM (10mL), was added a solution of isocyanate (**6**) (920 g, 0.93 mmol), DIPEA (0.2 mL, 0.93 mmol) and DMAP (22 mg, 0.17 mmol) in a mixture of DCM/DMF (1:1) and the mixture was shaken overnight. The solution was drained and the resin was

washed with DMF (3×20 mL), DCM (3×20 mL) and MeOH (3×20 mL) and ether (3×20 mL). The coupling reaction was monitored by a quantitative ninhydrin test.

Dde deprotection

To the resin (200 mg, 0.32 mmol), pre-swollen in DCM (5mL), was added 2% hydrazine in DMF (3 mL) and the reaction mixture was shaken for 2 h. The solution was then drained and the resin was washed with DMF (3×20 mL), DCM (3×20 mL) and MeOH (3×20 mL). The coupling reaction was monitored by a quantitative ninhydrin test.

8-(9-Fluorenylmethyloxycarbonyl-amino)-3,6-dioxaoctanoic acid (Fmoc-PEG-OH) coupling

A solution of Fmoc-PEG-OH (3.0 mmol, 10 eq) in DMF (3 mL) and Oxyma (3.0 mmol, 10 eq) was added and the mixture was stirred for 10 min. DIC (3.0 mmol, 10 eq) was then added and the resulting mixture was stirred for further 5 min. The solution was added to pre-swollen resin (**7**) in DCM and the reaction mixture was shaken for 6 h. The solution was drained and the resin was washed with DMF (3×10 mL), DCM (3×10 mL) and MeOH (3×10 mL). The coupling reaction was monitored by a quantitative ninhydrin test.

Fmoc deprotection

To the resin (pre-swollen in DCM) was added 20% piperidine in DMF (5 mL) and the reaction mixture was shaken for 10 min. The solution was drained and the resin was washed with DMF (3×10 mL), DCM (3×10 mL) and MeOH (3×10 mL). This procedure was repeated twice.

Peptide coupling

Peptide Sequence: Thr-Gly-Arg-Ala-Lys-Arg-Arg-Nle-Gln-Tyr-Asn-Arg-Arg

A solution of the appropriate Fmoc-amino acid (3.0 mmol, 10 eq) (Fmoc-Arg(Pbf)-OH, Fmoc-Asn(Trt)-OH, Fmoc-Tyr(tBu)-OH, Fmoc-Gln(Trt)-OH, Fmoc-Nle-OH, Fmoc-Lys(Boc)-OH, Fmoc-Ala-OH, Fmoc-Gly-OH, Fmoc-Thr(tBu)-OH) and Oxyma (3.0 mmol, 10 eq) was added and the mixture was stirred for 10 min. DIC (3.0 mmol, 10 eq) was then added and the resulting mixture was stirred for further 5 min. The solution was added to the pre-swollen resin in DCM and the reaction mixture was shaken for 6 h. The solution was drained and the resin washed DMF (3×20 mL), DCM (3×20 mL) and MeOH (3×20 mL). The coupling reactions were monitored by a quantitative ninhydrin test. Any reaction that had not gone to completion was repeated.

4-Chloro-7-nitrobenzofurazan (NBD-Cl) coupling

To a solution of NBD-Cl (3.0 mmol, 10 eq) in dry DMF (3mL) was added DIPEA (3.0 mmol, 10 eq). The resulting solution was added to resin, pre-swollen in DCM, and the reaction mixture was shaken

for 6 h. The solution was drained and the resin washed DMF (3×20 mL), DCM (3×20 mL) and MeOH (3×20 mL). The reaction was monitored by a quantitative ninhydrin test.

TFA cleavage and purification of NBD-UBI$_{dend}$

The resin (800 mg), pre-swollen in DCM, was treated with a cleavage cocktail of TFA:phenol:water:TIS (88:5:5:2, 8 mL) for 3 h. The solution was drained and the resin was washed with the cleavage cocktail (~2ml) and the solutions combined and concentrated *in vacuo*. The crude material was dissolved in a minimum amount of the cleavage cocktail (500 µL) and added to ice-cold ether (7.5 mL). The precipitated solid was collected by centrifugation and the solvent removed by decantation and the precipitate was washed with cold ether (3×5 mL). The precipitate was then purified by preparative reverse phase HPLC and the required fractions were pooled and lyophilized to afford NBD-UBI$_{dend}$.

NBD-UBI$_{dend}$ Characterization

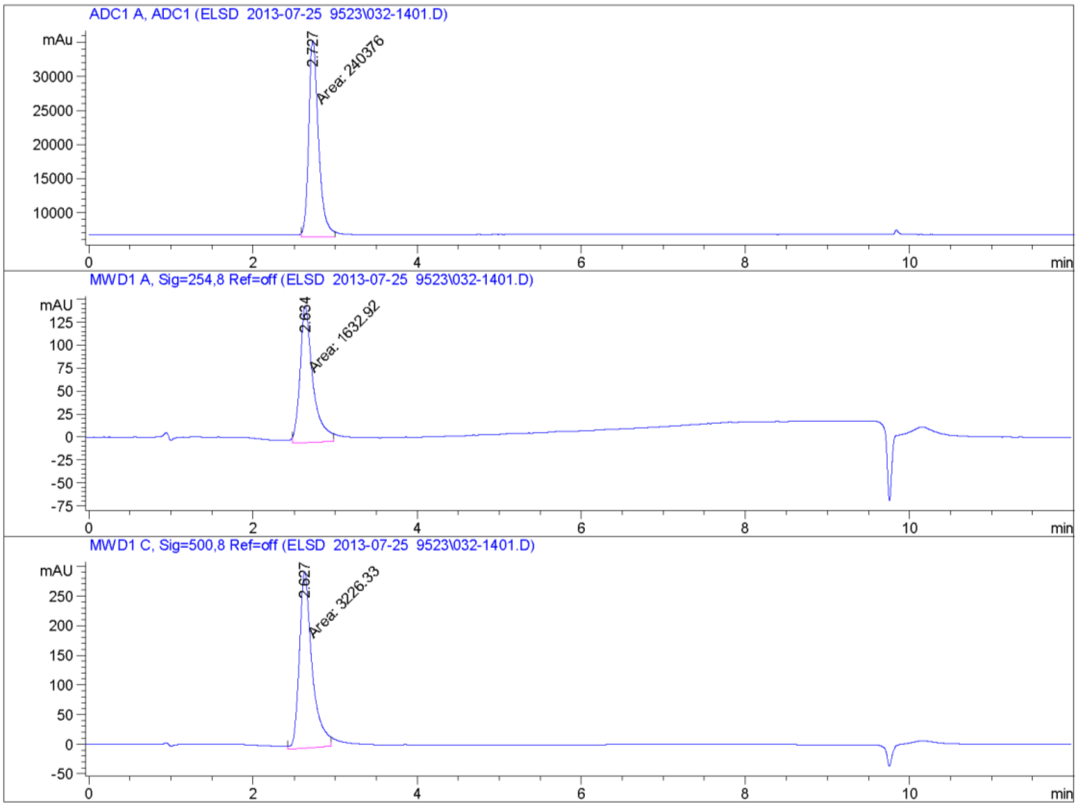

Figure M1. HPLC chromatogram of the purified NBD-UBI$_{dend}$.

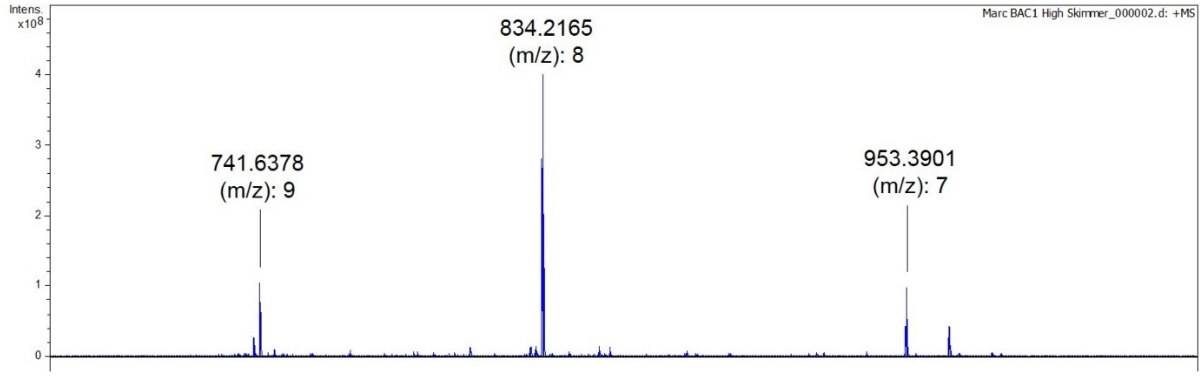

Figure M2. HRMS spectra of NBD-UBI$_{dend}$. Calculated for $C_{275}H_{465}N_{113}O_{82}$: 741.7616 (*m/z*: 9), 834.3008 (*m/z*: 8), 953.3425 (*m/z*: 7); found: 741.6378 (*m/z*: 9), 834.2165 (*m/z*: 8), 953.3901 (*m/z*: 7).

Supplementary references

1. N. Avlonitis, M. Debunne, T. Aslam, N. McDonald, C. Haslett, K. Dhaliwal, M. Bradley, Highly specific, multi-branched fluorescent reporters for analysis of human neutrophil elastase. *Organic & biomolecular chemistry* **11**, 4414-4418 (2013).
2. E. Kaiser, R. L. Colescott, C. D. Bossinger, P. I. Cook, Color test for detection of free terminal amino groups in the solid-phase synthesis of peptides. *Analytical biochemistry* **34**, 595-598 (1970).
